# A New Distribution-Free Approach to Constructing the Confidence Region for Multiple Parameters

**DOI:** 10.1371/journal.pone.0081179

**Published:** 2013-12-04

**Authors:** Zhiqiu Hu, Rong-Cai Yang

**Affiliations:** 1 Department of Agricultural, Food and Nutritional Science, University of Alberta, Edmonton, Alberta, Canada; 2 Research and Innovation Division, Alberta Agriculture and Rural Development, Edmonton, Alberta, Canada; Pennsylvania State University United States of America

## Abstract

Construction of confidence intervals or regions is an important part of statistical inference. The usual approach to constructing a confidence interval for a single parameter or confidence region for two or more parameters requires that the distribution of estimated parameters is known or can be assumed. In reality, the sampling distributions of parameters of biological importance are often unknown or difficult to be characterized. Distribution-free nonparametric resampling methods such as bootstrapping and permutation have been widely used to construct the confidence interval for a single parameter. There are also several parametric (ellipse) and nonparametric (convex hull peeling, bagplot and HPDregionplot) methods available for constructing confidence regions for two or more parameters. However, these methods have some key deficiencies including biased estimation of the true coverage rate, failure to account for the shape of the distribution inherent in the data and difficulty to implement. The purpose of this paper is to develop a new distribution-free method for constructing the confidence region that is based only on a few basic geometrical principles and accounts for the actual shape of the distribution inherent in the real data. The new method is implemented in an R package, distfree.cr/R. The statistical properties of the new method are evaluated and compared with those of the other methods through Monte Carlo simulation. Our new method outperforms the other methods regardless of whether the samples are taken from normal or non-normal bivariate distributions. In addition, the superiority of our method is consistent across different sample sizes and different levels of correlation between the two variables. We also analyze three biological data sets to illustrate the use of our new method for genomics and other biological researches.

## Introduction

Confidence interval estimates of individual parameters are more informative than simple point estimates and thus they are widely used in statistical inference [1], [2], [3]. However, a joint confidence region (CR) for two or more parameters is often needed in practical applications. Classical applications include the joint CR for two or more regression coefficients in a typical multiple regression analysis [2]. More recently, there have been calls for the use of the joint CRs to ascertain superior genotypes identified for target environments in biplot analysis of genotype-by-environment interaction [4], [5] or to unambiguously infer about population stratification in human admixtures [6], [7], [8], [9], [10].

Construction of the confidence intervals or regions for parameters often assumes that the data are from a normal distribution and they are balanced. For example, for bivariate normally-distributed data, the required CR is an ellipse whose shape depends largely on the level of the correlation between the two variables. However, when the distribution is unknown or hard to be characterized, several nonparametric procedures are available for construction of the confidence intervals or regions. Data peeling is a valuable approach to inspecting the structure of multivariate data [11]. The predominant implementation of data peeling is based on the convex hull of the data [12]. In convex hull peeling, the outmost convex hull is identified, the observations in the convex are assigned with index value of one and then these observations are removed from the data. This procedure is iterated but the index value is increased by one for each iteration until all observation are assigned with indexes. A CR can be determined by identifying the layer of peeling with the indexes higher than the threshold (preset significant level). The peeling approach is further developed by considering data depth [13], [14] to address the inquiry to the effectiveness of the procedure [11], [15]. HPDregionplot [16] is another nonparametric method for constructing CR. The fundamental behind the HPDregionplot is to use the contour that embraces the desired proportion of the capacity based on the two-dimensional kernel density estimates [17] as CR.

One of the key limitations with these parametric and non-parametric methods is the inaccurate estimation of the coverage rate by the CRs with the data of unknown distributions. All the non-parametric methods are computationally demanding [18] and some of them (e.g., HPDregionplot) are sensitive to small sample sizes. In this paper, we introduce a simple distribution-free geometry-based procedure that allows for constructing the CR for two or more parameters when there is no knowledge about the sampling distributions of the estimated parameters. We examine statistical properties of the new method through computer simulations and illustrate its use through two biological examples.

## Materials and Methods

### Quantile for a single parameter

For a single parameter, the distribution-free approach to computing a percentile is quite straightforward. Although different definitions for percentiles exist [19], all the definitions would lead to similar results given a large number of the random samples [20]. After obtaining estimates from individual random samples, three basic steps are followed to construct a distribution-free confidence interval: (1) to sort the 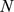 estimates in the ascending order; (2) to search for the nearest ranks for 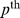 percentile by picking up the closest integers to 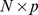; and (3) to estimate the desired percentile by linear interpolation between the two consecutive ranks.

### Quantiles for multiple parameters

Although the above procedure considers one variable only, it can be extended to the calculation of the CR simultaneously for two or more variables. For simplicity, let us consider the case of two variables. Let 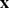 and 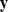 be the two vectors of size (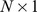). The values in vector 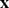 are the Euclidean distances, in geometry, between the observed points and the vertical coordinate (i.e., the reference line at 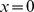). Similarly, the values in vector **y** are the Euclidean distances between the observed points and the horizontal coordinate (i.e., the reference line at 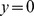). Thus the quantiles estimated for a single parameter are also the quantiles of the relative distances between the observed points and the reference line at 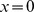 or 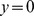. However, with unknown joint sampling distribution of variables 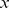 and 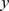, all potential reference lines across the entire plane need to be considered while constructing the distribution-free CR.

**Figure 1:**
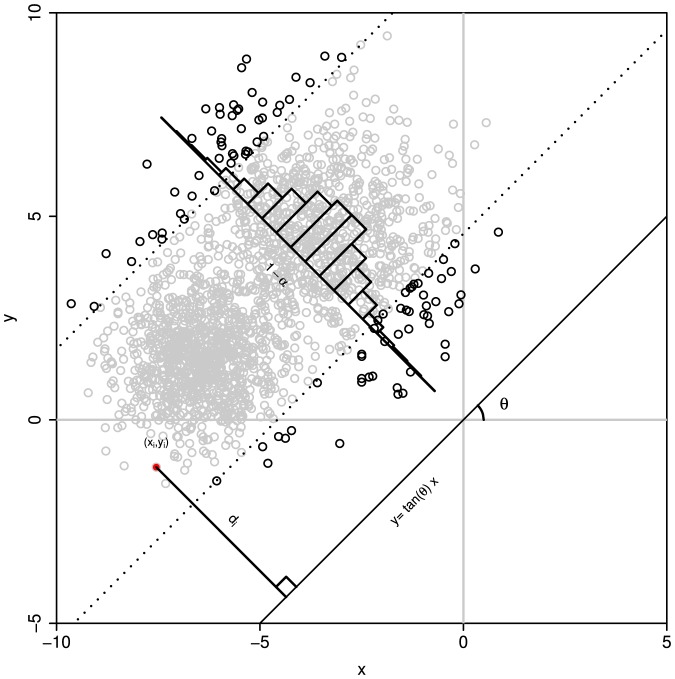
The confidence region constructed for an arbitrary reference line. The simulated population is an equal-proportional mixture of the observations sampled from two bivariate normal distributions which are given by 
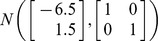
 and 
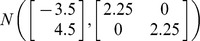
, respectively. The two parallel dashed lines are the boundaries of the confidence region for the reference line with angle 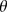. The black and gray open circles are points outside and within the boundaries, respectively. The histogram shows the distribution of the distances with respect to the reference line and the heights of the bars are the observed frequencies multiplied by 5.

Here we describe a general geometry-based approach to constructing the CR for any bivariate data. As mentioned earlier, the confidence interval for one variable can be regarded as a special case in which the reference line has been set to either vertical or horizontal coordinate axis (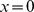 or 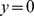). Now let us consider the confidence interval for an arbitrary reference line (cf. Figure 1). Since the positions of the observations in relation to a reference line, i.e., the distances with directions, are used to obtain the percentile, all reference lines have the same slopes but with different intercepts. We simplify the derivation by assuming all reference lines through the origin of the coordinates. The arbitrary reference line is expressed as
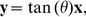
(1)where 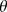 is the angle between the reference line and the horizontal abscissa (see section A of Appendix S1 for detailed derivation). It is also evident from Figure 1 that the relative position (distance) of the 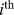 observations (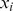, 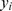) to the reference line as given in eq (1), is calculated as (see section A of Appendix S1 for detailed derivation),
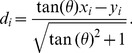
(2)


Applying eq. (2) repeatedly for all 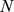 observations, we obtain the relative positions that are stored in vector 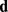. If the 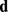 vector is viewed as a single variable, then the algorithm described earlier can be directly applied to calculate the required quantiles. Here we consider that the statistical inference is based on the two-tailed tests. For a specified significance level 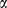, the confident interval of a single parameter is flanked by the observed lower- and upper- boundaries, i.e., the 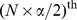 and 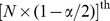 percentiles. In geometry view, the boundaries 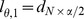 and 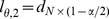 represent the distances between two parallel lines and the reference line to ensure that 95% of the total data points lie within the boundaries and 5% outside the boundaries in the direction 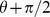 (see Figure 1). The function of the 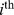 boundary line in an arbitrary direction in the plane is given as (see section B of Appendix S1 for detailed derivation)
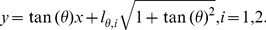
(3)


Let us denote the subset of all out-of-boundary points in the direction with the angle of θ as 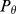. The observed significant level in this direction is expected to approximate the specified significant level for a single parameter (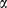), 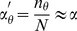
(4)where 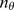 is the number of out-of-boundary points in the direction with the angle of θ in 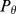. Using the same strategy, we obtain the boundary lines in all directions by rotating the reference line in all directions over the plane. By taking all boundaries jointly into consideration, we construct a CR as a polygon in the plane under the assumption that the significant level for each direction is 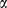. To the newly constructed region, the observations outside the polygon are counted as
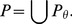
(5)


Since the directions with angles of 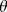 and 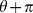 are actually the same reference line, we require the slope of the reference lines to increase monotonically with the angle while rotating the reference line with the range of 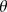 being 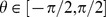.

**Figure 2:**
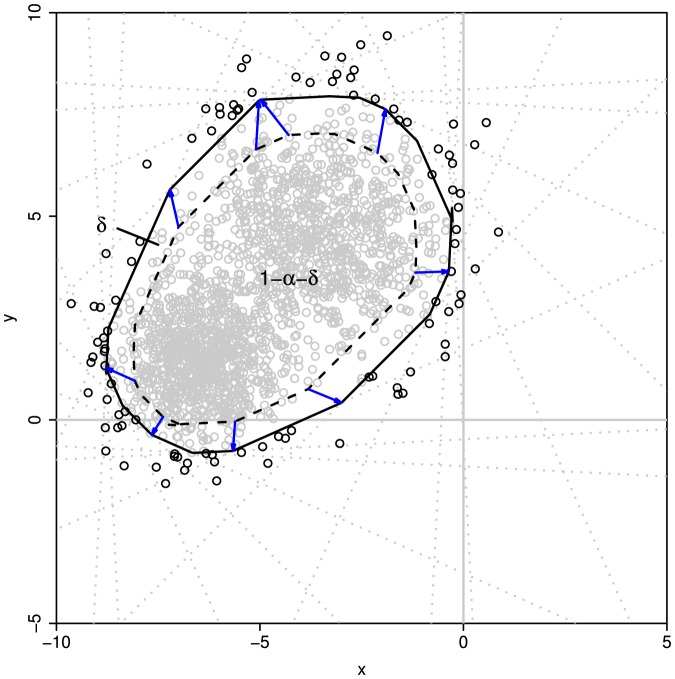
The confidence region and polygon boundaries obtained by rotating the reference line. The polygon is formed by the dash lines and the confidence region is constructed by setting the significant level for each test at 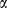. The outer polygon with solid lines represents the expanded confidence region with observed significant level approximating to desired significant level 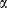.

It should be noted that the method described above can also be viewed as a set of multiple tests and thereby the observed significant level for the CR is actually greater than the 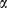 level that is specified for each test, i.e., 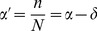
(6)where 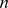 is the number of observations in 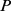, 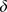 is the difference between the expected and the desired significant levels (Figure 2). Thus, the 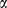 value that is actually specified to calculate the CR for each test should be lower than the desired significant level for multiple tests. Although it is difficult to provide a general function to describe the relationship between the two values, the desired 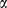 value can be obtained iteratively from the follow equation
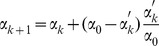
(7)where 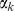 is the assigned value of the significant level required for generating the CR in each direction, 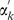 denotes the actually significant level for the CR bounded by the polygon as showed in eq (6), and 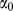 is the desired significant level for the overall test.

In this study, we construct the CR that is approximated by a polygon in a two-dimensional plane for the two variables. In each direction, the polygon is bounded by the lower- and upper-boundaries as given in eq (3). The vertices of the polygon are the crossover points of all adjacent boundary lines. The vertice between two adjacent reference lines with the angle of δ is a point in the plane whose two coordinate values are given by, 
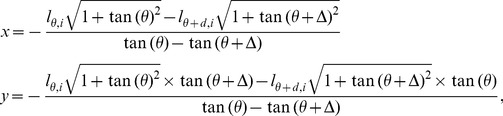
(8)where, 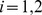 (see section C of Appendix S1 for detailed derivation).

## Results

### Simulation studies

The performance of our new method is evaluated by analyzing simulation data. We simulate bivariate data with two variables 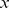 and 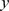. Three bivariate sampling distributions are considered in our simulations. In simulation I, 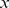 and 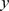 are sampled from a bivariate normal distribution 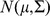, where 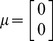 and 
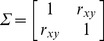
 with 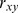 being the correlation between variables,*x* and *y*. In simulation II, the two variables (*x* and *y*) are generated from a bivariate noncentral *F*-distribution following the approach of Song and Hsiao [21]. The marginal *F*-distribution of each of the two variables is specified as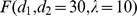, where 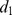 and 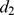 are degrees of freedom and *λ* is the noncentrality parameter. In simulation III, the two variables (*x* and *y*) are generated from a mixture of two bivariate normal distributions which is given by 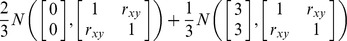. In all three simulations, the correlation 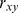 takes three values of 0, 0.5 and 0.9. In each simulation, we take *n* = 200 and *n* = 10,000 pairs of *x*−*y* observations from the distribution to represent small and large samples, respectively.

For each data, empirical CRs are constructed using our new method (distfree.cr/R, http://statgen.ualberta.ca), the classical ellipsoidal confidence region approach [2] implemented by the CAR package [22] in R [23] and other three nonparametric methods, the HPDregionplot in the emdbook/R package [16], the classic convex hull peeling [12], and data peeling based on the Tukey’s depth [24]. The CRs are constructed for seven significance levels, 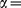
0.005, 0.01, 0.025, 0.05, 0.1, 0.2 and 0.5. However, only one level of significance 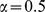 is used for the peeling approach based on the Tukey’s depth because we use the bagplot approach [24], via the bagplot function in the aplpack/R package [25], to implement the peeling based on Tukey’s depth, but both the method [24] and the software implementation [25] are developed exclusively for 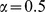 (Dr Peter Wolf, private communication). We develop an R code to implement the classical convex hull peeling approach based on its definition (available at http://statgen.ualberta.ca). The adequacy of the CRs is measured using coverage discrepancy plots [26] for each simulation run, i.e., the deviation of the realized-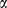
estimate of each method to its real value. The realized-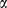
is calculated as the proportion of the observations outside an empirical confidence polygon, which is determined using the pnt.in.poly function in the SDMTools/R [27].

**Figure 3:**
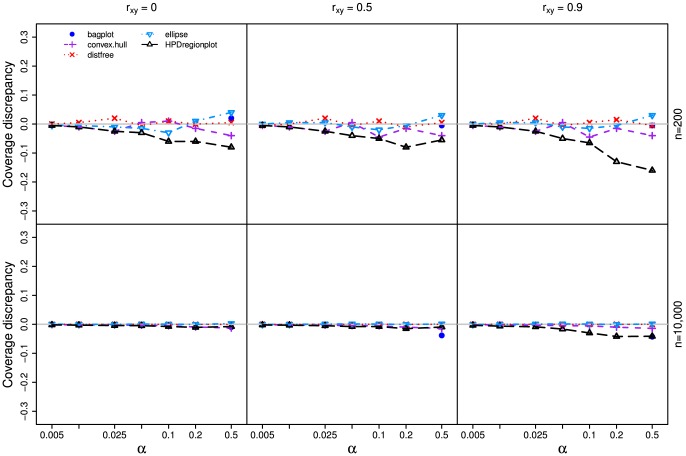
Coverage discrepancy of the empirical confidence regions in simulation design I. The empirical confidence regions for a range of probability levels (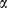 = 0.005 to 0.5) are constructed by five methods (distfree, ellipse, bagplot, convex hull peeling and HPDregionplot) based on a small sample (n = 200) and a large sample (n = 10,000) taken from a bivariate normal distribution with mean vector 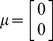 and variance-covariance matrix of 
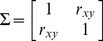
, where *r_xy_* takes 0, 0.5 and 0.9. The confidence region by bagplot is available only at 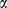 = 0.5.

**Figure 4:**
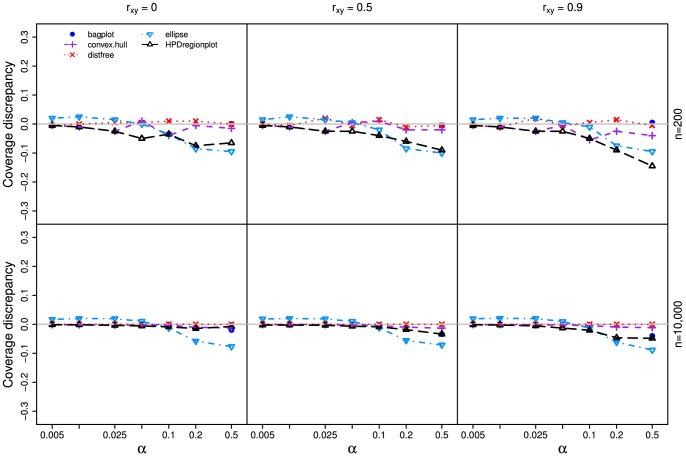
Coverage discrepancy of the empirical confidence regions in simulation design II. The empirical confidence regions for a range of probability levels (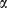 = 0.005 to 0.5) are constructed by five methods (distfree, ellipse, bagplot, convex hull peeling and HPDregionplot) based on a small sample (n = 200) and a large sample (n = 10,000) taken from a bivariate noncentral *F*-distribution with the correlation between two variables of *r_xy_*  =  0, 0.5 and 0.9. The confidence region by bagplot is available only at 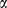 = 0.5.

**Figure 5:**
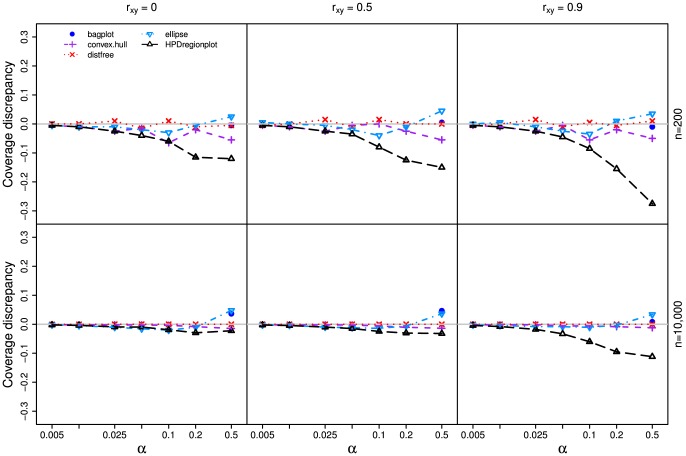
Coverage discrepancy of the empirical confidence regions in simulation design III. The empirical confidence regions for a range of probability levels (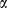 = 0.005 to 0.5) are constructed by five methods (distfree, ellipse, bagplot, convex hull peeling and HPDregionplot) based on a small sample (n = 200) and a large sample (n = 10,000) taken from a mixture of two bivariate normal distributions which is given by 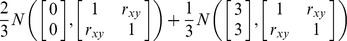, where *r_xy_* takes 0, 0.5 and 0.9. The confidence region by bagplot is available only at 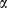 = 0.5.

**Figure 6:**
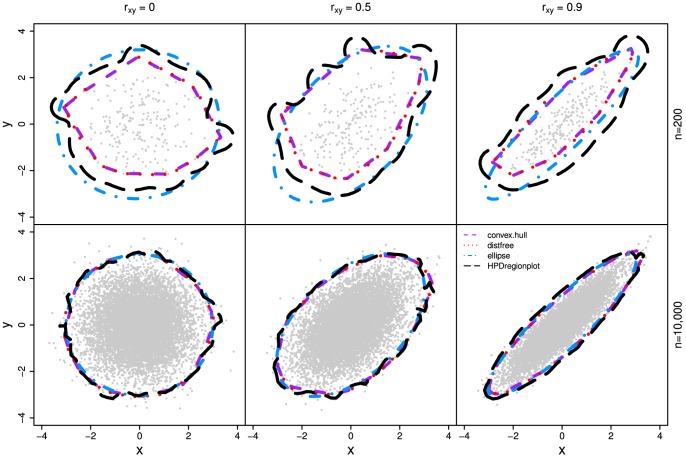
The 95% empirical confidence regions estimated by the four methods (distfree, ellipse, convex hull peeling and HPDregionplot) in simulation I which is detailed in Figure 3.

**Figure 7:**
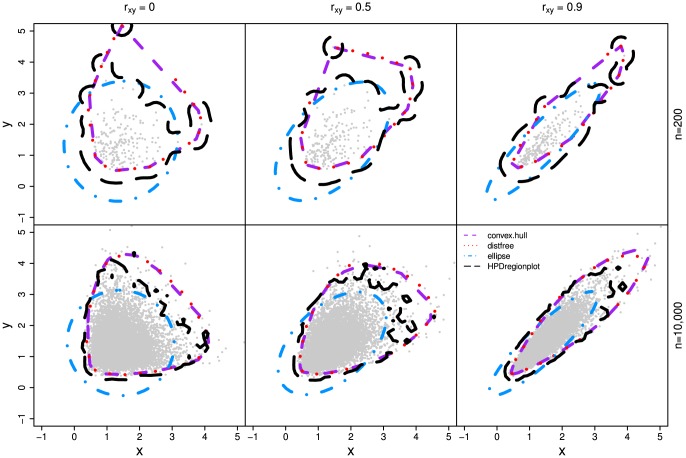
The 95% empirical confidence regions estimated by the four methods (distfree, ellipse, convex hull peeling and HPDregionplot) in simulation II which is detailed in Figure 4.

**Figure 8:**
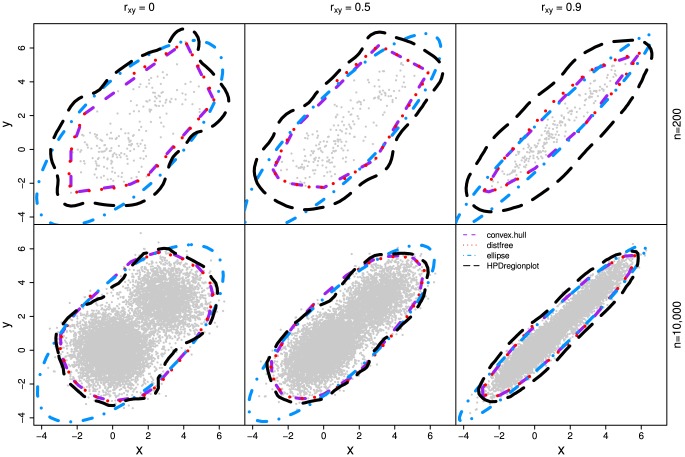
The 95% empirical confidence regions estimated by the four methods (distfree, ellipse, convex hull peeling and HPDregionplot) in simulation III which is detailed in Figure 5.

In all three simulations, our method outperforms other methods (Figures 3, 4, and 5) as the realized-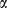 estimates by our method is close to or coincides with the true significance levels for both small (n = 200) and large (n = 10,000) samples with all three 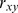 values. The classic ellipsoidal method provides overestimation when 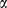 is low and underestimation when 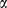 is high. All methods including the ellipsoid approach produce similar 95% CRs for the data from the bivariate normal distribution as in simulation I (Figure 6). However, the CRs determined by the ellipsoid approach fail to account for the actual shapes of non-normal sampling distributions as in simulations II and III (Figures 7 and 8). The HPDregionplot is the most sophisticated strategy in capturing the shape of non-normal sampling distribution in all simulations. However, the realized-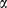 estimates by the HPDregionplot approach are constantly lower than the true significance levels; the underestimation tends to increase with the significant level and the correlation (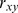), and it is more pronounced for non-normal data in simulations II (Figure 4) and III (Figure 5) than for normal data in simulation I (Figure 3). It is somewhat surprising to note that the bagplot method performs as well as our method with small sample (n = 200) but it performs poorly with the large sample (n = 10,000) particularly when 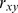 is high.

### Empirical examples

We also analyze three empirical examples to illustrate the use of our new method for the analysis of real data sets. The first data set is taken from Table 4.3 of Rawlings et al. [2]. Since the data set was already described and analyzed by Rawlings et al. [2], we will only recapitulate the essential details of the data. The original data set consisted of physical fitness measurements on 31 men involved in a physical fitness program at the North Carolina State University. The variables measured were age (years), weight (kg), oxygen uptake rate (ml per kg body weight per minute), time to run 1.5 miles (minutes), heart rate while resting, heart rate while running (at the same time oxygen uptake was measured), and maximum heart rate while running. Rawlings et al. [2] carried out the multiple regression analysis to investigate the response of oxygen uptake to the change of time to run 1.5 miles (minutes), heart rate while resting, heart rate while running (at the same time oxygen uptake was measured), and maximum heart rate while running.

**Figure 9:**
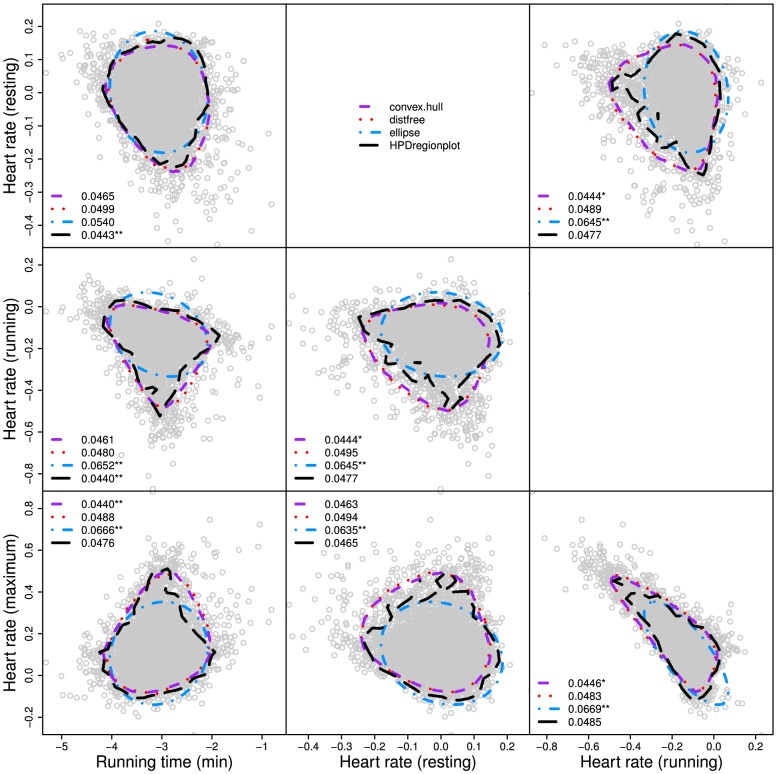
The joint confidence regions of the regression coefficient estimates of the physical fitness measurements on 31 men involved in a physical fitness program at the North Carolina State University. The numbers in the figure are the realized-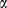 values of the corresponding confidence regions. * and ** indicate significant deviations of the realized-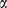
values from 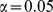, according to chi-square tests, at *P*<0.05 and *P*<0.01, respectively.

For illustration, we only show the CRs of the pairwise regression coefficients as constructed by our new method and the classic methods. The CRs are constructed using the convex hull data peeling approach [12], the classical ellipsoidal method as implemented using the CAR package in R [22], the HPDregionplot in the emdbook/R package [16] and our new geometry-based method (distfree.cr/R, http://statgen.ualberta.ca). Bootstrapping is used to generate 10,000 random samples from the original data. The size of each bootstrap sample is set to 31, the number of individuals as used in the original study. The multiple regression analysis is done for each bootstrap sample. The pairwise regression coefficients as well as their CRs (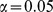) calculated by the four approaches are plotted (Figure 9). The realized-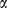
values are calculated as the proportions of the total observations that lie outside the CRs determined by our new method and the classical methods for all six pairs of regression coefficients. For each pair, the chi-square test statistics is computed to examine the significance of coverage discrepancies of the empirical CRs under the preset significance level of 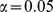. The testing results show the superiority of our new method over the classic methods because the deviations of the realized-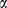
values from 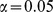 by our new method are not biased from 0.05 in all pairs whereas there are 4, 6, and 2 pairs with biased realized-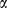 estimates for convex hull peeling, ellipse, and HPDregionplot, respectively.

**Figure 10:**
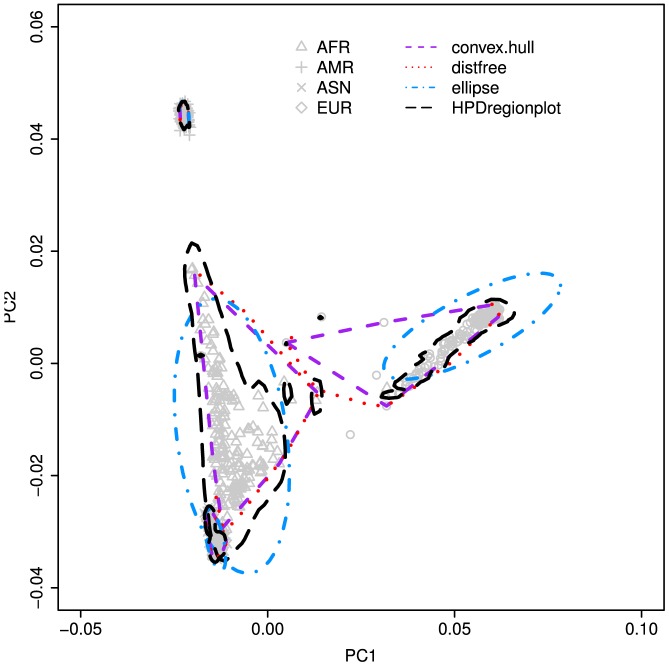
Plots of 1,092 human individuals in 2-D space using the scores of the first two principal components as calculated by EIGENSTRAT based on 51,529 SNP markers. The polygons represent the 95% confidential regions of four individual populations: AFR for African, AMR for Ad Mixed American, ASN for East Asian, and EUR for European.

The second data set is obtained from the 1000 Genomes project [28]. This data set consists of 1,092 human individual records from four super populations, which include 246 Africans (AFR), 181 Ad Mixed Americans (AMR), 286 East Asians (ASN), and 379 Europeans (EUR). For each record, there is an integrated haplotype map of 38 million single nucleotide polymorphisms (SNPs), 1.4 million short insertions and deletions and 14,000 larger deletions. Prior to the analysis, we use the PLINK software [29] to remove the SNPs with minor allele frequency (MAF) of <0.05 and the SNPs with interval sizes smaller than 50 k base pairs in order to have a manageable subset of data. After the removal, a total of 51,529 SNPs remain and we use this subset of the data for the subsequent analysis. Principal component analysis (PCA) as implemented in the EIGENSTRAT software [9] is carried out. The first two principal components are used to generate the scatter plots as well as to construct the 95% confidential regions for individual super populations using the new method as well as the classical methods (Figure 10).

It is evident from Figure 10 that the four methods generate distinctly different CRs particularly for the AFR and AMR populations. The four methods also reveal different patterns of population differentiation. The CRs constructed by the ellipse and HPDregionplot methods suggest that the EUR population is largely contained within the AMR population. In contrast, the CRs constructed by our new method and convex hull peeling approach suggest that the EUR population is somewhat distinguishable from the AMR population. In addition, the realized-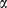 values derived from our new methods are always closer to the prescribed significance level of 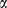 = 0.05 than those from the classical methods.

The third empirical example is the winter wheat (*Triticum aestivum* L.) data set that has been used (e.g., Yan et al.[30]) for the biplot analysis of genotype×environment interaction. We (Yang et al. [31] and Hu and Yang [32]) have recently analyzed this data set as well to illustrate the application of our bootstrapping approach to statistical inference about genotypic and environmental scores obtained from singular value decomposition (SVD) of the two-way genotype×environment table. Here the example serves to show how the CRs constructed for individual genotypic and environmental scores corresponding to the first two principal components (PC1 and PC2) are valuable in pointing out the uncertainty around the mega-environments delineated by the earlier studies. Briefly, the data set consists of the yields of 18 winter wheat genotypes (G1 to G18) tested at nine environments (E1 to E9) in Ontario, Canada. Prior to the analysis, the deviations of cell means for all 162 (18×9) genotype-environment combinations from location means are calculated. The resultant matrix is the basis for bidirectional bootstrapping, SVD and Procrustes rotation as explained in Hu and Yang [32].

**Figure 11:**
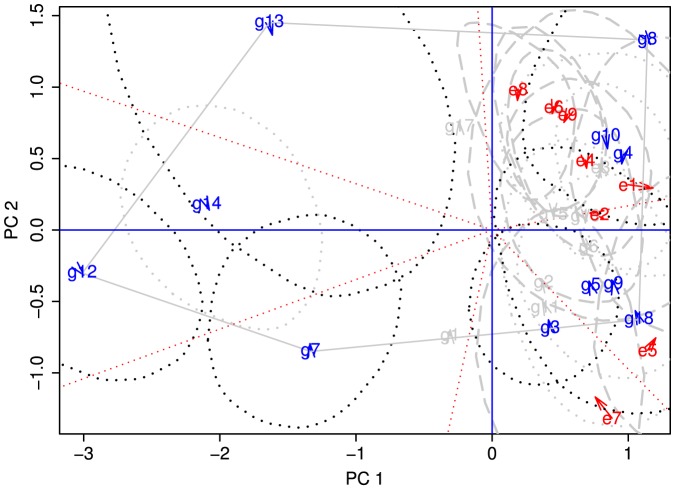
Biplot of 18 genotypic scores and nine environmental scores from the Ontario winter wheat data. The 95% confidence regions are constructed for the genotypic and environmental scores using 10,000 bootstrap samples.

The biplot of PC1 vs. PC2 genotypic and environmental scores along with the 95% CR is presented in Figure 11. The PC1 and PC2 account for about 78% of the total variability. To highlight key features in the biplot, the CR are displayed only for those scores that are significantly different from the origin of the biplot [i.e., the CR of the scores that do not include the point of (0,0)]. A hexagon is drawn to connect six genotypes (G3, G7, G8, G12, G13 and G18) that are located at the corners (i.e., vertices) of the hexagon in the biplot. To further facilitate the interpretation of the biplot, six line segments perpendicular to different sides of the polygon are drawn through the origin to subdivide the polygon into six sectors involving different subsets of environments and genotypes: the genotype at the corner of each sector is considered as the ‘best’ performer in the environments included in that sector as often claimed in the earlier studies (e.g., Yan et al. [30]). However, it is evident from the 95% CR of the scores that the ‘best’ genotypes are often not statistically different from other genotypes. For example, genotype G8 at the upright corner is indistinguishable from genotypes G4 and G10 in the same sector, judging from their overlapped CR. Simple visual inspection of the biplot [30] claimed that genotype G18 yielded more than genotype G8 in eastern Ontario (represented by E5 and E7) and G8 yielded more than G18 in southwestern Ontario (represented by the other seven environments). With the 95% CR being now attached to individual scores (Figure 11), this claim is no longer true because the CRs for G8 and G18 overlap. Thus, identification of superior genotypes or mega-environments based on the initial inspection of biplots is simply a curious visual observation only and it must be substantiated by subsequent parametric or non-parametric statistical assessments before being recommended for practical utility.

## Discussion

In this study, we develop a new geometry-based, distribution-free approach to constructing the CR for two or more variables. Our new method is based only on a few basic geometrical principles and accounts for the actual shape of the distribution (Figures 1 and 2). Thus, it should be a significant complement to the existing parametric (ellipsoidal [2]) and nonparametric methods including bagplot [16], convex hull peeling [12], and HPDregionplot [25]).

Our method outperforms other parametric and non-parametric approaches to constructing CRs judging from coverage discrepancy plots of realized-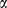 estimates. It is evident from Figures 3, 4, and 5 that our method always provides more accurate estimates of 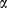 than the other methods regardless of whether the sampling distribution is normal (simulation I) or not (simulations II and III). In addition, the superiority of our method is consistent over different levels of correlation between the two variables. So why is our method better? Simply put, it is the only method that accommodate for the actual shape of the distribution and allows for adjusting the realized-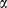
value to an individual data point level. While the convex hull peeling and data peeling based on Tukey’s depth can also account for the shape of the actual distribution represented by the original data, the realized-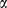
value may still be different from the true 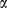 because the CR is determined by a ‘peeling’ layer. Thus, all the data points on the same layer have to be included or excluded simultaneously once the layer is determined as the border of the CR. The true 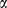 value can be under- or over-estimated unless each peeling layer consists of only one data point, an unlikely scenario for not too small samples or unless, by chance, the peeling layer along with outer layers constitute the exact 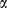 value.

The realized-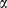 estimates by the parametric ellipsoidal method and semi-parametric HPDregionplot may also be biased, but for a different reason. In these methods, the original data are used merely to estimate parameters. It is these estimated parameters along with assumed normal distribution, rather than the original data that are used for constructing CRs. If the data is normally distributed, an unbiased estimate of 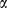 can be achieved; if, on the other hand, the data is from a non-normal distribution, the estimate of 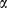 may be biased upward or downward. If the true CR is a concave polygon or a crescent moon or the union of disjoint convex areas, then the HPDregionplot is the *only* method that is capable of capturing the true shape of the CR(e.g., the shape of the simulated distribution in simulation III). However, the HPDregionplot may produce the CRs with multiple isolated polygons for small sample sizes (e.g., simulation II for n = 200). Furthermore, in the current version of the emdbook/r package (version 1.3.2.1) on CRAN [16], the HPDregionplot function may also generate unclosed rather than closed polygons for CRs. In an attempt to address this issue, Dr. Ben Bolker, the author of the emdbook/r package, provided us with a set of new parameters for HPDregionplot function (private communication). While the use of these new parameters guarantees the closed polygons by extending the regions for the kde2d function, the polygons derived by the new HPDregionplot function are slightly larger than that calculated by the previous version, thereby leading to the underestimation of the realized-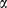 values. Unfortunately, there is currently no solution to the issue. The HPDregionplot approach works well with accurate estimates of the empirical kernel density. High information content in the original data would be especially important for accurate estimation. This is probably why higher correlation between the two variables has caused greater discrepancy between the realized and true 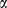 values (Figures 3, 4, and 5). However, no similar trend is observed when the autocorrelation within the variables is considered (Figures S1-S4).

As shown above, the coverage discrepancy is a necessary criterion for evaluating the performance of different methods for constructing CRs. Nevertheless, it is not a sufficient criterion. For example, it is evident from Figure 4 that, in simulation II, the realized 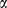 estimates by the ellipsoidal method are biased upward with low 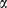, but downward with high 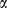. An inflexion point exists near 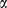 = 0.05 where there is little coverage discrepancy. However, this coincidence does not necessarily mean that the ellipsoidal-based CR can be used to approximate the CR for the sample taken from an F-distributed data because there is bias at all other 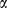 levels. It is shown (Figure S5) that the point of the transition from over- to under-estimation of 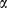 changes with the degrees of freedom for the F-distributions, but there is little dependence on the noncentrality parameter.

Since each curve in the coverage discrepancy plot (Figures 3, 4, and 5) is calculated from a single random sample, the repeatability of the coverage discrepancy patterns revealed by the plots may be questioned [26]. To confirm the results in Figures 3, 4, and 5, ten additional random samples are generated from the three simulated bivariate distributions described earlier. The coverage discrepancy curves by the five methods are displayed in Figure S6. The plots show that the patterns revealed by the coverage discrepancy curves are fairly stable across different samples.

We provide detailed descriptions of our new distribution-free approach to constructing CR for two parameters only. This does not mean that it works only for the two-dimensional data. In fact, our method can be extended to higher-dimension situations. In constructing a CR for three or more parameters, we need to calculate the distances between the data points and reference planes (three variables) or reference hyperplanes (four or more variables). For example, the formula for the distance between the *i*th point in the three-dimensional space 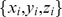 and the reference plane (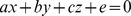) is given by Korn and Korn [33],
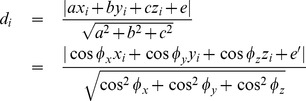
(9)


The second part of equation (9) is obtained using the ‘normal’ form of the reference plane (a normal line is the line perpendicular to the reference plane),
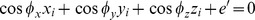
where
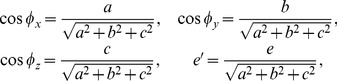
(10)with *φ_x_*, *φ_y_* and *φ_z_* being the angles between the normal line and axis 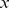, axis 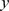 and axis 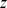, respectively, and 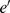 being the distance between the reference plane and the origin. The actual implementation requires the following two considerations: (1) the sample size required to construct a reliable CR is exponentially increased with the addition of variables; and (2) the amount of computation under higher dimension circumstances is escalating as more reference lines need to be taken into account while constructing the high-dimensional CR. Nevertheless, further research is needed for implementing and interpreting the multidimensional CRs.

Although the normal distribution has been widely assumed in the past [1], [2], the joint sampling distribution of the pairwise regression coefficients that are obtained from the data of the oxygen intake experiment by bootstrapping is evidently deviated from a bivariate normal distribution (Figure 9). Thus the basic assumption required for constructing ellipsoidal CRs may often be incorrect and this might lead to distorted CRs and thus to incorrect practical uses.

The second empirical example serves to demonstrate the use of our new method for adding the statistical inference capability to one of the most popular tools currently used in human population genomics. The correction for population stratification is an essential step towards eliminating spurious genetic effects in the genome-wide association study (GWAS) of admixed populations [34]. Cavalli-Sforza et al. [6] proposed the use of the principal component analysis (PCA) for detecting the stratification among human populations. Recently, the strategy has been further developed and adopted in using genomic data for the analysis of population stratification in human [7], [8], [9], [10]. The effectiveness of such PCA-based detection depends on correct inference about the ancestry and population structure. Currently, the commonly used means of inferring the population stratification is the use of scatter plots of the first few principal components known as "radiation of circular or elliptic clines from a specification area" or the "principal-component map" [6]. However, the determination of population sharing or membership based on these plots or maps is somewhat arbitrary because it is based solely on visual inspection. Since the sampling distributions of the principal component scores derived from SNP markers are unknown, the use of the classical ellipsoidal method for constructing the CRs may not be adequate. The third example shows further utility of our new method for strengthening the biplot analysis of genotype×environment interaction. Thus, our distribution-free approach to constructing any multivariate CRs provides a statistical basis for such determination.

## Supporting Information

Figure S1
**The impact of autocorrelations (0, 0.5 and 0.9) on the coverage discrepancy plots for small sample** 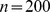 **in simulation I which is detailed in** **Figure 3** **.**
(EPS)

Figure S2
**The impact of autocorrelations (0, 0.5 and 0.9) on the coverage discrepancy plots for large sample** 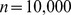 **in simulation I which is detailed in** **Figure 3** **.**
(EPS)

Figure S3
**The impact of autocorrelations (0, 0.5 and 0.9) on the coverage discrepancy plots for small sample** 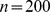 **in simulation II which is detailed in** **Figure 4** **.**
(EPS)

Figure S4
**The impact of autocorrelations (0, 0.5 and 0.9) on the coverage discrepancy plots for large sample** 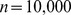 **in simulation II which is detailed in** **Figure 4** **.**
(EPS)

Figure S5
**The effect of F distribution with equal degrees of freedom (** 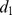 **=** 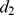 **) on the coverage discrepancy plots of the empirical confidence regions as approximated by a normal distribution.**
(EPS)

Figure S6 **Coverage discrepancy plots based on 10 independent simulated samples of sizes n = 200 and n = 10,000. The two variables are assumed independent.**
(EPS)

Figure S7 **The confidence region constructed for two reference lines.**
(EPS)

Appendix S1 **Derivation of equations.**
(DOCX)
