# Supplementary material for: A New Distribution-Free Approach to Constructing the Confidence Region for Multiple Parameters
Source: PLoS One. 2013 Dec 4;8(12):e81179. doi: 10.1371/journal.pone.0081179 (PMC3852254; doi:10.1371/journal.pone.0081179)
Supplement: Appendix S1 — Derivation of equations. (DOCX) [file pone.0081179.s008.docx]

# A: Derivation of equations (1) and (2)

Straight line in a Cartesian plane can be described algebraically by linear equation. In the case of two dimensions, the equation for the line is often written in a general form as given by Coxeter [[1](#_ENREF_1)]:

$ax+by+c=0$,

where *a*, *b* and *c* are real constants with $a\neq0$ and $b\neq0$. It can be expressed in the more similar slope-interception form:

$$y=mx+c'$$

where $m=-\frac{a}{b}$ is the slope of the line and $c'=-\frac{c}{b}$ is the y-intercept of the line. An obvious example of eq is the linear regression equation. Given the angle $\theta$ between a line and the horizontal abscissa (x-axis), the slope of the line can be described geometrically as a tangent function (tan) of $\theta$, i.e., $m=\tan(\theta)$. For a line through the origin, there is zero intercept (i.e., $c'=0$), eq is reduced to

$y=\tan(\theta)x$,

which is equal to eq (1) in the text.

The general formula for the distance from a point ($x_{i},y_{i}$) to a line is well known (e.g., [[2](#_ENREF_2)]),

$$d_{i}^{'}=\text{distance}\left(ax+by+c=0, \{\mathrm{x}_{i}, y_{i}\}\right)=\frac{|ax_{i}+by_{i}+\mathrm{c}|}{\sqrt{a^{2}+b^{2}}}$$

When eq is rewritten in the form of eq , $\tan(\theta)x-y+0=0$, we have,

$\left\{\begin{matrix} a=\tan(\theta) \\ b=-1 \\ c=0 \end{matrix}\right.$.

Thus, $d_{i}$ in eq can be obtained by substituting the values of $a$, $b$, and $c$ in eq ,

$d_{i}^{'}=\frac{\left|\tan(\theta)x_{i}-y_{i}\right|}{\sqrt{\tan(\theta)^{2}+1}}$.

The distance ($d_{i}^{'}$) in eq has no direction. The direction of the distance is determined by the signed distance function as explained in Delfour and Zolesio [[3](#_ENREF_3)],

$d_{i}=\left\{\begin{matrix} \frac{\left|\tan(\theta)x_{i}-y_{i}\right|}{\sqrt{\tan(\theta)^{2}+1}} & \text{for} \tan(\theta)x_{i}\geq y_{i} \\ -\frac{\left|\tan(\theta)x_{i}-y_{i}\right|}{\sqrt{\tan(\theta)^{2}+1}} & \text{for}\tan(\theta)x_{i}<y_{i} \end{matrix}\right.$,

which is equivalent to

$d_{i}=\frac{\tan(\theta)x_{i}-y_{i}}{\sqrt{\tan(\theta)^{2}+1}}$.

This provides a derivation of eq (2) in the main text. In eqs and , a negative distance ($d_{i}<0$) is obtained if the point ($x_{i},y_{i}$) is located at the upper side of the line (or the left side if the line is vertical) and a positive value ($d_{i}\geq0$) is obtained if it is at the opposite side of the line. The eq is used to calculate point-line distances in this study.

# B: Derivation of equation (3)

Assume there are two parallel lines ($L_{1}$ and $L_{2}$), $ax+by+c_{1}=0$ and $ax+by+c_{2}=0$). The distance between the two lines is known [[1](#_ENREF_1)]

$$d'=\frac{\left|c_{2}-c_{1}\right|}{\sqrt{a^{2}+b^{2}}}$$

If $L_{1}$ through the origin, i.e., $c_{1}=0$, and given the $a$ and $b$ values in eq , the above equation is rewrite as

$$d'=\frac{\left|c_{2}\right|}{\sqrt{\tan(\theta)^{2}+1}}$$

Thus, the y-intercept of the line $L_{2}$ is,

$$c_{2}=\pm d'\sqrt{\tan(\theta)^{2}+1}$$

In other words, there are two potential $L_{2}$ lines that are parallel to $L_{1}$,

$$\left\{\begin{matrix} y=\tan(\theta)x+d'\sqrt{1+\tan(\theta)^{2}} & L_{2} \text{is above} L_{1} \\ y=\tan(\theta)x-d'\sqrt{1+\tan(\theta)^{2}} & L_{2} \text{is below} L_{1} \end{matrix}\right.$$

Replacing the distance (d’) in eq with the signed distance ($d$) defined in eq , we have,

$$y=\tan(\theta)x+d\sqrt{1+\tan(\theta)^{2}}$$

Let $l_{\theta,1}=d_{N\times\alpha/2}$ and $l_{\theta,2}=d_{N\times(1-\alpha/2)}$ be the distances from the reference line to the lower-boundary and upper- boundary as determined by the $(N\times\alpha/2)^{\text{th}}$ and $[N\times(1-\alpha/2)]^{\text{th}}$ percentiles, respectively. Therefore, the $i$th boundary line in the direction $\theta+\pi/2$ is given by,

$$y=\tan(\theta)x+l_{\theta,i}\sqrt{1+\tan(\theta)^{2}}, i=1,2$$

This proves eq (3) in the main text.

# C: Derivation of equation (8)

Assume that there is an intersection ($x_{0}$, $y_{0}$) between two lines ($L_{1}$ and $L_{2}$), $y=m_{1}x+c_{1}^{'}$ and $y=m_{2}x+c_{2}^{'}$. at this intersection point, we have

$$\begin{matrix} m_{1}x_{0}+c_{1}^{'}=m_{2}x_{0}+c_{2}^{'} \\ m_{1}x_{0}-m_{2}x_{0}=c_{2}^{'}-c_{1}^{'} \\ x_{0}=\frac{c_{2}^{'}-c_{1}^{'}}{m_{1}-m_{2}}=-\frac{c_{1}^{'}-c_{2}^{'}}{m_{1}-m_{2}} \end{matrix}$$

Then, $y_{0}$ can obtained by substituting the $x$ in $L_{1}$ by the $x_{0}$ from eq ,

$$\begin{matrix} y_{0}=m_{1}x+c_{1}^{'} \\ =m_{1}\frac{c_{2}^{'}-c_{1}^{'}}{m_{1}-m_{2}}+c_{1}^{'} \\ =m_{1}\frac{c_{2}^{'}-c_{1}^{'}}{m_{1}-m_{2}}+\frac{m_{1}-m_{2}}{m_{1}-m_{2}}c_{1}^{'} \\ =\frac{m_{1}c_{2}^{'}-m_{1}c_{1}^{'}+m_{1}c_{1}^{'}-m_{2}c_{1}^{'}}{m_{1}-m_{2}} \\ =\frac{m_{1}c_{2}^{'}-m_{2}c_{1}^{'}}{m_{1}-m_{2}}=-\frac{c_{1}^{'}m_{2}-c_{2}^{'}m_{1}}{m_{1}-m_{2}} \end{matrix}$$

Assuming that the angels for lines$L_{1}$ and $L_{2}$ are $\theta$ and $\theta+\Delta$ (Appendix figure S7), respectively, we rewrite the equations of two lines in the form of eq . The slopes and y-intersects for lines $L_{1}$ and $L_{2}$ are

$$\begin{matrix} m_{1}=\tan(\theta) \\ c_{1}^{'}=l_{\theta,i}\sqrt{1+\tan(\theta)^{2}} \\ m_{2}=\tan(\theta+\Delta) \\ c_{2}^{'}=l_{\theta+\Delta,i}\sqrt{1+\tan(\theta+\Delta)^{2}} \end{matrix}$$

The coordinates of the interaction between the two boundary lines are obtained by replacing $m_{1}$, $c_{1}^{'}$, $m_{2}$, and $c_{2}^{'}$ in the eqs and ,

$$\begin{matrix} x_{0}=-\frac{l_{\theta,i}\sqrt{1+\tan(\theta)^{2}}-l_{\theta+d,i}\sqrt{1+\tan(\theta+\Delta)^{2}}}{\tan(\theta)-\tan(\theta+\Delta)} \\ y_{0}=-\frac{l_{\theta,i}\sqrt{1+\tan(\theta)^{2}}\times\tan(\theta+\Delta)-l_{\theta+d,i}\sqrt{1+\tan(\theta+\Delta)^{2}}\times\tan(\theta)}{\tan(\theta)-\tan(\theta+\Delta)} \end{matrix}$$

This proves eq (8) in the main text.

# Reference

1. Coxeter HSM (1969) Introduction to geometry. New York,: Wiley. 469 p.

2. Deza MM, Deza E (2013) Encyclopedia of Distances. Berlin Heidelberg: Springer-Verlag. 650 p.

3. Delfour MC, Zolesio JP (1994) Shape Analysis via Oriented Distance Functions. Journal of Functional Analysis 123: 129-201.
